# Comparison of linear and nonlinear programming approaches for “worst case dose” and “minmax” robust optimization of intensity‐modulated proton therapy dose distributions

**DOI:** 10.1002/acm2.12033

**Published:** 2017-03-13

**Authors:** Maryam Zaghian, Wenhua Cao, Wei Liu, Laleh Kardar, Sharmalee Randeniya, Radhe Mohan, Gino Lim

**Affiliations:** ^1^ Office of Performance Improvement The University of Texas MD Anderson Cancer Center Houston Texas USA; ^2^ Department of Radiation Physics The University of Texas MD Anderson Cancer Center Houston Texas USA; ^3^ Department of Radiation Oncology Mayo Clinic Arizona Phoenix Arizona USA; ^4^ PROS Inc Houston Texas USA; ^5^ Department of Industrial Engineering University of Houston Houston Texas USA

**Keywords:** intensity‐modulated proton therapy (IMPT), linear programming (LP), nonlinear programming (NLP), robust optimization, robustness evaluation

## Abstract

Robust optimization of intensity‐modulated proton therapy (IMPT) takes uncertainties into account during spot weight optimization and leads to dose distributions that are resilient to uncertainties. Previous studies demonstrated benefits of linear programming (LP) for IMPT in terms of delivery efficiency by considerably reducing the number of spots required for the same quality of plans. However, a reduction in the number of spots may lead to loss of robustness. The purpose of this study was to evaluate and compare the performance in terms of plan quality and robustness of two robust optimization approaches using LP and nonlinear programming (NLP) models. The so‐called “worst case dose” and “minmax” robust optimization approaches and conventional planning target volume (PTV)‐based optimization approach were applied to designing IMPT plans for five patients: two with prostate cancer, one with skull‐based cancer, and two with head and neck cancer. For each approach, both LP and NLP models were used. Thus, for each case, six sets of IMPT plans were generated and assessed: LP‐PTV‐based, NLP‐PTV‐based, LP‐worst case dose, NLP‐worst case dose, LP‐minmax, and NLP‐minmax. The four robust optimization methods behaved differently from patient to patient, and no method emerged as superior to the others in terms of nominal plan quality and robustness against uncertainties. The plans generated using LP‐based robust optimization were more robust regarding patient setup and range uncertainties than were those generated using NLP‐based robust optimization for the prostate cancer patients. However, the robustness of plans generated using NLP‐based methods was superior for the skull‐based and head and neck cancer patients. Overall, LP‐based methods were suitable for the less challenging cancer cases in which all uncertainty scenarios were able to satisfy tight dose constraints, while NLP performed better in more difficult cases in which most uncertainty scenarios were hard to meet tight dose limits. For robust optimization, the worst case dose approach was less sensitive to uncertainties than was the minmax approach for the prostate and skull‐based cancer patients, whereas the minmax approach was superior for the head and neck cancer patients. The robustness of the IMPT plans was remarkably better after robust optimization than after PTV‐based optimization, and the NLP‐PTV‐based optimization outperformed the LP‐PTV‐based optimization regarding robustness of clinical target volume coverage. In addition, plans generated using LP‐based methods had notably fewer scanning spots than did those generated using NLP‐based methods.

## 1 Introduction

Intensity‐modulated proton therapy (IMPT) is potentially one of the most effective ways to treat cancer because it can deliver highly conformal and homogenous dose distributions to a target with a complex shape while maximally sparing adjacent healthy tissues.^1^ It is delivered using thin scanning beams (beamlets) of protons with a sequence of discrete energies. For a given energy, the dose from a proton beam or beamlet increases as a function of depth of penetration in the patient until it reaches a peak (the Bragg peak) and then falls sharply to near zero. The high potential of IMPT owes to the fact that protons have a finite range and a sharp dose falloff at the end of the range and that IMPT can control the range (energy) and intensity of individual beamlets. For IMPT to be effective, a high degree of precision and accuracy in delivery is required so that the dose distribution that is actually delivered is a good approximation of the dose distribution in the treatment plan.^2^, ^3^, ^4^, ^5^, ^6^, ^7^

Unfortunately, the characteristics of protons that make them suitable for radiotherapy also make them sensitive to various types of uncertainty. The two most important sources of uncertainty in IMPT are the beam range and patient setup uncertainties. These uncertainties can result in deviation of the delivered IMPT dose distribution from the planned distribution, which may lead to suboptimal treatment decisions and unforeseen outcomes. Therefore, these uncertainties must be considered during IMPT plan optimization.

In photon therapy, the conventional approach to handling patient setup uncertainties and organ motion is to expand the clinical target volume (CTV) by an empirically determined margin to form a planning target volume (PTV). The underlying assumption in the determination of the CTV‐to‐PTV margin is that the CTV will be sufficiently covered with high probability (e.g., 95%) in the face of uncertainties. This approach works well for photon therapy because the variations in photon dose distributions when patient anatomy changes are relatively small.^7^, ^8^ However, for IMPT, uncertainties can cause substantial perturbations in the dose distributions not only in the CTV‐to‐PTV margins but also within the CTV as well as in regions distal and proximal to the target al.ong the beam paths. Dose distributions in normal tissues lateral to the CTV also may be substantially perturbed, especially when protons pass through complex heterogeneities. Thus, simply applying the concept of PTV to proton therapy cannot efficiently mitigate the impact of uncertainties, so alternative approaches to PTV‐based optimization are required.^9^

One such approach is robust optimization, which aims to produce optimal, resilient IMPT plans in the face of uncertainties. Researchers have conducted several probabilistic and scenario‐based studies to incorporate uncertainties into IMPT plan optimization.^2^, ^3^, ^4^, ^5^, ^6^, ^7^, ^8^, ^9^ In a probabilistic approach, the expectation value of the random objective function is optimized.^5^, ^6^ Three scenario‐based approaches have been proposed, that is, the “worst case dose”,^10^ “minmax,”^9^ and “composite objective” 11 robust optimization. In reality, they are all worst case approaches. The first is based on the worst case dose in each voxel. The second considers the worst case value of the objective function for the dose distribution as a whole. The third takes the worst case value of the objective function for the dose distribution in each structure. All of these scenario‐based approaches can work with a linear programming^12^ or nonlinear programming (NLP)^7^ model. Some groups have proposed a worst case dose robust optimization approach using an LP model to consider range uncertainties,^5^, ^13^ whereas Pflugfelder et al. proposed a worst case dose distribution‐based robust optimization approach using a nonlinear quadratic objective function.^4^ This approach also can be used with linear objective functions. Motivated by Pflugfelder and colleagues,^4^ Liu et al. developed a modified nonlinear worst case dose distribution‐based robust optimization approach that additionally penalized hot spots within the target for better target dose homogeneity.^7^

Fredriksson et al. first proposed minmax robust optimization. This nonlinear constrained model does not assume a probability distribution for uncertainties.^9^ Chen et al. also reported on a multicriteria minmax optimization approach using a piecewise‐linear convex constrained model similar to that proposed by Fredriksson and colleagues.^8^

To date, only a handful of studies have compared different IMPT robust optimization approaches. Fredriksson generalized a class of robust optimization methods, including expected value and minmax optimization.^14^ He studied and compared special cases and found that the minmax approach had advantages over other methods in controlling hot spots within the target and sparing the organs at risk (OARs). More recently, Fredriksson and Bokrantz compared three approaches to NLP‐based worst case dose optimization.^11^ However, they did not identify a dominant broadly applicable approach. They observed identical behavior of plan quality and robustness in all three approaches without any conflicting planning criteria but clear differences in the presence of conflicting criteria.

In all of these studies, researchers investigated robust optimization techniques for IMPT using either an LP or NLP model. However, a comparison of the performance of LP and NLP models with one or more robust optimization approaches has yet to be reported. Therefore, we performed this study to identify and outline differences in the behavior of various LP‐ and NLP‐based approaches and models for IMPT robust optimization. To that end, we developed and evaluated worst case dose (voxel‐wise) and minmax methods for both LP and NLP formalism models. To better understand the influence of LP‐ and NLP‐based models on robust optimization results, we also performed PTV‐based conventional optimization with both LP and NLP objective functions. Thus, we compared six optimization methods: LP‐PTV‐based, NLP‐PTV‐based, LP‐worst case dose, NLP‐worst case dose, LP‐minmax, and NLP‐minmax. Specifically, we compared the plan optimality and robustness and number of scanning spots (surrogate of plan efficiency) for the plans created with each of the six methods. Although Fredriksson and Bokrantz previously compared quadratic worst case dose and minmax approaches to account for setup uncertainties for two patients with prostate cancer, the performance of different methods may depend on the treatment site.^11^ Therefore, in our study, we compared the six methods for two patients with prostate cancer, a patient with a skull base tumor, and two patients with head and neck cancer. In addition, we considered both beam range and patient setup uncertainties.

## 2 Materials and methods

### 2.A Patient data, beam configurations, and uncertainty scenarios

The relative performance of various robust optimization approaches was evaluated by regenerating treatment plans for two patients with prostate cancer, one with skull base cancer, and two with head and neck cancer, all of whom had undergone proton therapy at our institution. Two lateral fields were used for prostate cancer cases, whereas three fields were used for the other three cases. For each patient, eight uncertainty scenarios were assumed: two setup uncertainty scenarios (±5 mm for prostate cancer and ±3 mm for the other cancers) in the x, y, and z directions and two range uncertainty scenarios (±3.5% of the nominal range of the beams). For each patient, the six optimization methods described above and below were used to account for range and setup uncertainties. The PTV was chosen as the target for the conventional optimization approach and, appropriately, the CTV was used as the target for the robust optimization approaches.^7^, ^9^ The prescribed doses, target volumes, beam angles, dose grid resolutions, and margins used for the robust optimization approaches are listed in Table 1. All beams were coplanar (couch angle, 0 °).

**Table 1:** Dose and beam configurations and uncertainty scenarios for the robust optimization approaches used to generate treatment plans for each patient in our study. RBE denotes relative biological effectiveness

Case	Prescribed dose (Gy [RBE])	Target volume (cm^3^)	Gantry angles (°)	Dose grid resolution (mm)	Range uncertainty (%)	Setup error (mm)
1.Prostate cancer	76	43.44	90, 270	2.5	±3.5	±5
2.Prostate cancer	78	69.82	90, 270	2.5	±3.5	±5
3.Skull base cancer (chordoma)	74	20.90	75, 270, 300	2.5	±3.5	±3
4.Head and neck cancer (chordoma)	34	45.77	60, 290, 320	2.5	±3.5	±3
5.Head and neck cancer (nasopharynx)	70	20.12	70, 285, 290	2.5	±3.5	±3

### 2.B Optimization methods

For each patient, three optimization approaches were evaluated: conventional PTV‐based, worst case dose robust, and minmax robust optimization. For each approach, LP‐ and NLP‐based optimization models were developed.

#### 2.B.1 Conventional PTV‐based optimization

In conventional optimization, uncertainties were accounted for by expanding the CTV to a PTV via margins and treating the PTV as the target. The PTV was formed via isotropic expansion of 5 mm for prostate cancer and 3 mm for the other types of cancer to account for setup uncertainties. Cao et al. described details of the LP‐based optimization model, and Liu *et al*. described details of the NLP‐based optimization model.^7^, ^12^ Note that we consider only 3D IMPT technique in this study and scanning spots from all irradiation beams are simultaneously optimized.

#### 2.B.2 Worst case dose robust optimization

For a voxel inside the target, the minimum dose of the voxel of all dose distributions corresponding to different uncertainty scenarios was selected. For any voxel outside the target, the maximum dose of the voxel was selected. This formed the worst case dose distribution as follows:^2^, ^3^

$$\begin{array}{c} D_{i}=\min_{r}\left\{\sum_{j}d_{ij}^{r}\cdot x_{j},\;\forall i\in Target\right\}, \end{array}$$

$$\begin{array}{c} D_{i}=\max_{r}\left\{\sum_{j}d_{ij}^{r}\cdot x_{j},\;\forall i\notin Target\right\}, \end{array}$$

Where $d_{ij}^{r}$ is the influence matrix, which denotes the dose contributed by the *J* ^*th*^ beamlet per unit weight, and is received by voxel *i* under scenario *r*.

Robust optimization was performed by substituting the worst case dose distribution for the nominal dose distribution in each iteration. The decision variable *x*_*j*_ was the intensity of beamlet *j*. The optimization models applied to both CTV and OARs were as follows:

$$\mathrm{LP}:\min_{x}\left\{f\left(D_{i}\right)=\sum_{i}\lambda_{i}\left|D_{i}-D_{i}^{pres}\right||LB_{i}\leq D_{i}\leq UB_{i}\right\},$$

$$\mathrm{NLP}:\min_{x}\left\{f\left(D_{i}\right)=\sum_{i}\lambda_{i}{\left(D_{i}-D_{i}^{pres}\right)}^{2}\right\},$$

Where *λ*_*i*_ is the penalty weight, and $D_{i}^{pres}$is the prescription dose for each voxel. In linear programming, hard constraints are imposed on dose to each voxel, that is, ensuring that all constraints have to be satisfied otherwise no solution exists. *LB*_*i*_ and *UB*_*i*_ are lower and upper reference bounds on dose to each voxel. However, in nonlinear programming, only soft constraints are imposed. This means that the model penalizes deviations between delivered and prescribed doses, but does not require each voxel to meet certain dose limits.

#### 2.B.3 Minmax robust optimization

An alternative approach to worst case dose robust optimization is the minmax method described by Fredriksson et al.^9^ This method is designed to minimize the penalty of the worst case dose distribution scenario. Specifically, the objective function is evaluated under a number of treatment scenarios, and the worst calculated objective function is selected. In contrast with the worst case dose distribution, only physically realizable scenarios are considered. For this optimization approach, the dose distribution was calculated as $D_{i}^{r}=\sum_{j}d_{ij}^{r}\cdot x_{j},\forall i$.

$$\mathrm{LP}:\min_{x}\left\{\max_{r}\left\{f\left(D_{i}^{r}\right)=\sum_{i}\lambda_{i}|D_{i}^{r}-D_{i}^{pres}||LB_{i}\leq D_{i}\leq UB_{i}\right\}\right\}$$

$$\;\mathrm{NLP}:\;\min_{x}\left\{\max_{r}\left\{f\left(D_{i}^{r}\right)=\sum_{i}\lambda_{i}{\left(D_{i}^{r}-D_{i}^{pres}\right)}^{2}\right\}\right\}$$

Both CTV and OARs are incorporated in the optimization model. Relative strengths of the minmax and voxel‐wise robust optimization methods are described in the literature.^9^, ^11^

### 2.C Plan generation and comparison

Six IMPT plans were generated using the six optimization methods and compared in terms of plan quality and robustness and delivery efficiency. Only dose constraints (hard constraints for LP‐based models and soft constraints for NLP‐based models) were used in plan optimization. Both dose and dose‐volume constraints of nominal and worst case dose distributions were reviewed after optimization. If an optimized plan failed to meet such constraints, the plan could be re‐optimized by adjusting objective weights or dose constraints until all constraints were satisfied. Some of the key criteria for plan quality we used are listed below.

Prostate cancer:

1. Rectum: volume receiving a dose of 70 Gy (V70; relative biological effectiveness [RBE]) no greater than 20%.
2. Bladder: volumes receiving doses of 65 Gy (V65; RBE) and 40 Gy (V40; RBE) no greater than 25% and 50%, respectively.

Skull base and head and neck cancer:

1. Brainstem: maximum dose no greater than 60 Gy (RBE).
2. Spinal cord: maximum dose no greater than 45 Gy (RBE).
3. Oral cavity: mean dose no greater than 35 Gy (RBE).
4. Temporal lobe: volume receiving a dose of 60 Gy (V60; RBE) no greater than 1%.

For the sake of comparison, all plans were renormalized to cover at least 99% of the CTV by the prescribed dose in the nominal dose distribution. Dose‐volume histogram (DVH) indices (D_v_ and V_d_) were used to evaluate the quality of the plans. D_v_ denotes the amount of the dose received by more than v percent of the organ, and V_d_ denotes the percent volume of the organ receiving more than d Gy (RBE). D_v_ is proportional to the target coverage, whereas V_d_ is inversely proportional to OAR sparing. To illustrate differences in nominal plan quality and robustness among the six optimization methods, we selected several critical DVH indices for comparison for each cases. Those comparisons are discussed in the Results section.

To compare the robustness of the IMPT plans generated using the different methods, families of DVHs corresponding to different uncertainty scenarios were plotted along with the nominal DVHs. The resulting envelopes were used to assess the sensitivity of the plans under the uncertainty scenarios.^15^ The DVH‐family bandwidth method was also used to evaluate and compare the robustness of the different methods.^16^ The width of the DVH band (Δ) is inversely proportional to the robustness of the method. Δ(D_v_) denotes the width of the DVH band at volume v, and Δ(V_d_) denotes the width of the DVH band at dose d. This robustness evaluation technique effectively determined the robustness of the IMPT plans in the face of setup and range uncertainties. Next, to estimate the delivery efficiency of the plans, the resulting number of scanning spots with positive intensity generated using each method was assessed. Note that we used the same base scanning spot placement (e.g., same spot and layer spacing) for all optimization methods.

## 3 Results

**Figure 1:**
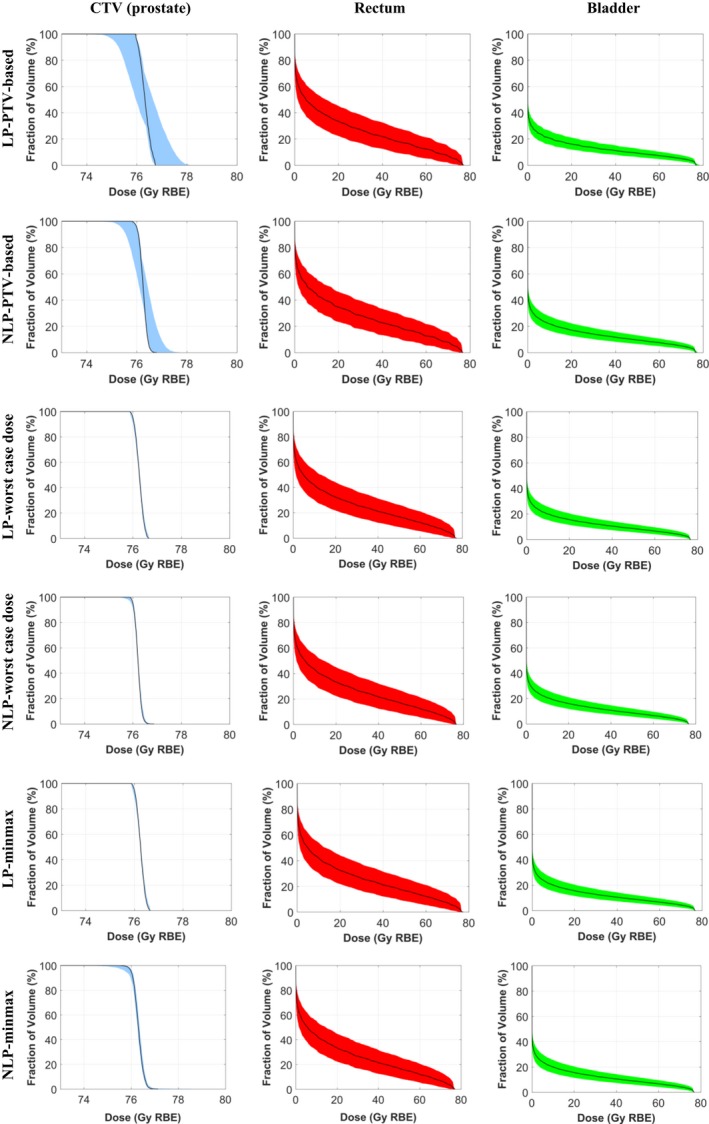
DVH bands for dose distributions covering all setup and range uncertainties for patient 1 (with prostate cancer) for the clinical target volume (CTV; left column), rectum (middle column), and bladder (right column), resulting from each of the six IMPT plan optimization methods. The width of the DVH band is inversely proportional to the robustness of the method. The solid lines indicate DVHs for the nominal dose distribution (i.e., without consideration of uncertainties).

We compared the plan quality and robustness of the six optimization strategies in terms of DVH indices for nominal doses and DVH band widths for uncertain scenario doses for five cancer patients. In addition, we compared the numbers of scanning spots after performing the six methods. Figure 1 displays the DVHs corresponding to the nominal dose distributions along with the DVH bands for one of the patients with prostate cancer (patient 1). In this representative case, CTV coverage with plans generated using the conventional PTV‐based optimization methods (Fig. 1, first two rows) was notably less robust than that with plans generated using other methods. Specifically, the DVH bands for the CTV were wider for PTV‐based optimization methods than for robust optimization methods, indicating that the plans generated using robust optimization were more robust regarding setup and range uncertainties than were those generated using conventional PTV‐based methods. Among the PTV‐based optimization methods, the NLP‐PTV‐based method outperformed the LP‐PTV‐based method in terms of robustness of CTV coverage. Furthermore, comparison of the robustness of the plans created using robust optimization (Fig. 1, bottom four rows) demonstrated that the worst case dose methods outperformed the corresponding minmax methods in covering the CTV. However, the robustness of normal tissue sparing was similar for all of the methods.

**Figure 2:**
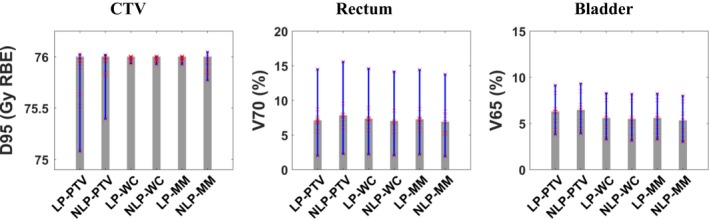
Dose statistics for nominal and uncertainty scenario dose distributions for patient 1 (with prostate cancer). The gray bars indicate the nominal doses. The red crosses indicate doses under different uncertainty scenarios. The blue lines show the band widths (i.e., ∆(D_v_), ∆(V_d_)) for different dose‐volume indices in DVH families under uncertainty scenarios.

To further evaluate plan robustness, we compared the DVH‐family band widths at key dose‐volume indices for the six methods for patient 1 (Fig. 2). For robust optimization, the LP‐based methods covered the CTV D95 slightly more robustly than did the corresponding NLP‐based methods. Using four robust optimization methods (LP‐worst case dose, NLP‐worst case dose, LP‐minmax, and NLP‐minmax), the NLP‐minmax plan was less robust for CTV D95 than were the other methods. However, the robustness for the rectum V70 and bladder V65 did not differ markedly among the four methods. In addition, the bladder V65 in nominal plans for the PTV‐based optimization methods was consistently improved by robust optimization.

**Figure 3:**
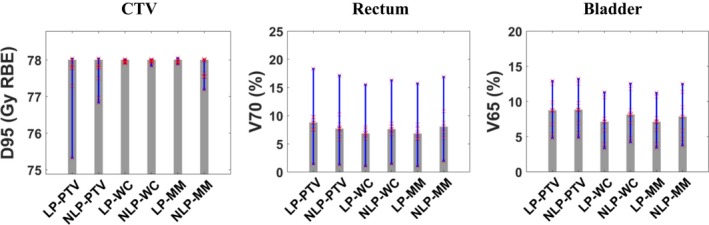
Dose statistics for nominal and uncertainty scenario dose distributions for patient 2 (a prostate cancer patient). The gray bars are based on the nominal doses. The red crosses are based on doses under different uncertainty scenarios. The blue lines show the band widths (i.e., ∆(D_v_), ∆(V_d_)) for different dose‐volume indices in DVH families under uncertainty scenarios.

DVH indices for nominal doses and DVH‐family band widths for patient 2 (the other prostate cancer patient) are shown in Fig. 3. The results of plan quality and robustness evaluation for this patient were consistent with those for the other prostate cancer patient. The robustness of CTV coverage for the PTV‐based optimization methods was inferior to that for the robust optimization methods. Among the PTV‐based optimization methods, NLP‐PTV‐based optimization was superior to the LP‐PTV‐based method in robustness of CTV coverage. Using four robust optimization methods, robustness of CTV D95 for the NLP‐minmax optimized plan was outperformed by other three robust optimization methods. We found no marked variations in the robustness of OAR (rectum and bladder) sparing among the different robust optimization methods. However, the rectum and bladder sparing with the NLP‐based methods was inferior to that with the LP‐based methods. Also, the robustness of CTV coverage for the worst case dose robust optimization methods was superior to that for the minmax methods. When comparing LP‐ and NLP‐based robust optimization methods, we observed that the CTV D95 robustness for the NLP‐based methods was inferior to that for similar LP‐based methods.

**Figure 4:**
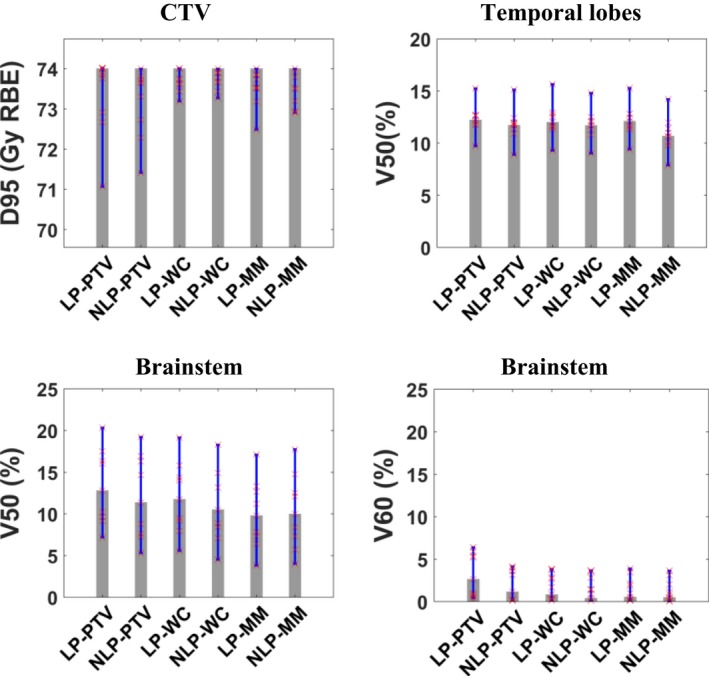
Dose statistics for nominal and uncertainty scenario dose distributions for patient 3 (with a skull base tumor). The gray bars are based on the nominal doses. The red crosses are based on doses under different uncertainty scenarios. The blue lines show the band widths (i.e., ∆(D_v_), ∆(V_d_)) for different dose‐volume indices in DVH families under uncertainty scenarios.

The robustness of CTV coverage for the PTV‐based methods was inferior to that for the robust optimization methods for patient 3 (the skull base cancer patient) (Fig. 4). In this case, CTV coverage robustness provided by the NLP‐based optimization methods was superior to that provided by the corresponding LP‐based methods. Moreover, worst case dose robust optimization methods generated plans with better CTV coverage robustness than did minmax methods. However, the robustness for the brainstem and temporal lobes in terms of variations in V50 and V60 was comparable for all optimization methods. The LP‐worst case dose method provided inferior brainstem sparing in terms of the V50 than did the other three robust optimization methods, whereas the NLP‐minmax method created a plan with superior temporal lobe sparing in terms of the V50.

**Figure 5:**
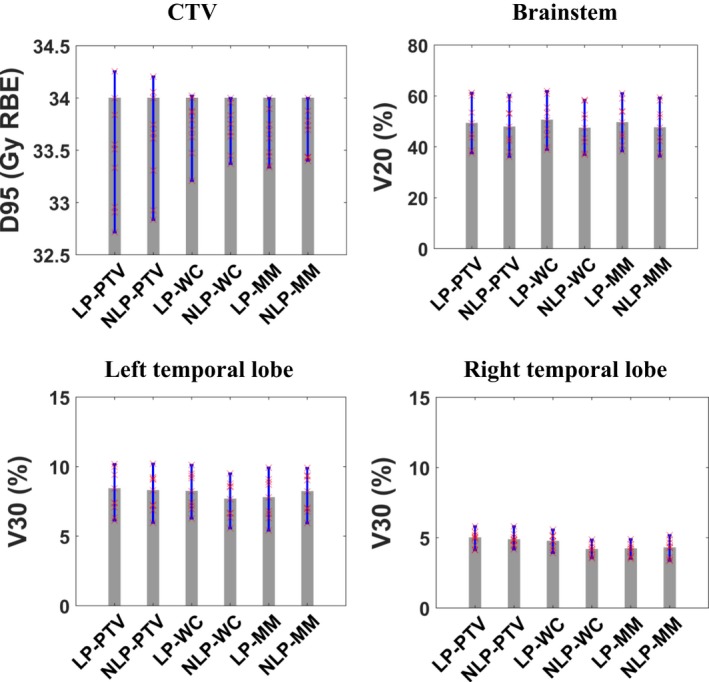
Dose statistics for nominal and uncertainty scenario dose distributions for patient 4 (with a head and neck tumor). The gray bars are based on the nominal doses. The red crosses are based on doses under different uncertainty scenarios. The blue lines show the band widths (i.e., ∆(D_v_), ∆(V_d_)) for different dose‐volume indices in DVH families under uncertainty scenarios.

For patient 4 (with head and neck cancer), the NLP‐based optimization methods were more robust in covering the CTV than were the LP‐based methods as shown in Fig. 5. The robustness of sparing critical structures was comparable for all robust optimization methods. Also, the PTV‐based methods clearly could not generate plans as robust in target coverage as those generated by the robust optimization methods.

**Figure 6:**
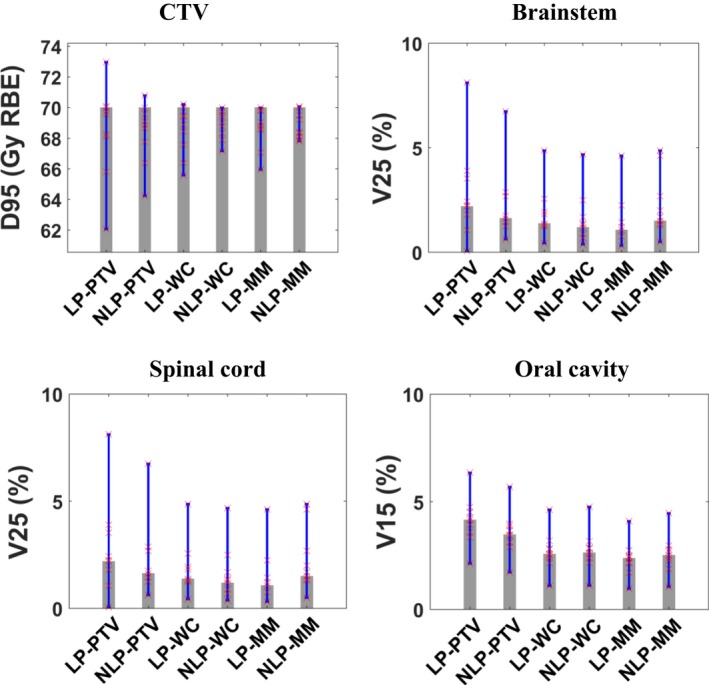
Dose statistics for nominal and uncertainty scenario dose distributions for patient 5 (with a head and neck tumor). The gray bars are based on the nominal doses. The red crosses are based on the doses under different uncertainty scenarios. The blue lines show the band widths (i.e., ∆(D_v_), ∆(V_d_)) for different dose‐volume indices in DVH families under uncertainty scenarios.

Figure 6 shows the dose statistics for patient 5 (a head and neck cancer patient). All of the robust optimization methods improved the plan robustness in CTV coverage and OAR (brainstem, spinal cord and oral cavity) sparing over that of the conventional PTV‐based optimization methods. With all PTV‐based, worst case dose, and minmax optimization approaches, the NLP‐based model outperformed the LP‐based model in providing robust CTV coverage. In terms of OAR sparing, no robust optimization method was consistently better than the others. The LP‐based model with conventional PTV‐based optimization provided brainstem and spinal cord sparing robustness in terms of the V25 inferior to that provided by the NLP‐PTV‐based model, but we found no marked variations in the robustness of OAR sparing among the different robust optimization methods.

In addition, we compared the PTV coverage for the six optimization methods. Table 2 lists the PTV D95 of optimized plans based on different methods for all five patient cases. It shows that both PTV‐based conventional and robust optimization methods were able to produce comparable PTV coverage.

**Table 2:** PTV D95 (Gy) for the six optimization approaches: LP‐ and NLP‐PTV, LP‐ and NLP‐WC, and LP‐ and NLP‐MM

Model	PTV D95 (Gy)				
	Patient 1	Patient 2	Patient 3	Patient 4	Patient 5
LP‐PTV	76.0	77.7	73.9	33.9	69.5
NLP‐PTV	76.0	77.5	73.7	33.7	69.7
LP‐WC	75.9	77.5	73.6	33.6	69.4
NLP‐WC	75.6	77.5	73.4	33.5	69.5
LP‐MM	75.7	77.5	73.5	33.4	69.3
NLP‐MM	75.6	77.4	73.6	33.5	69.4

**Figure 7:**
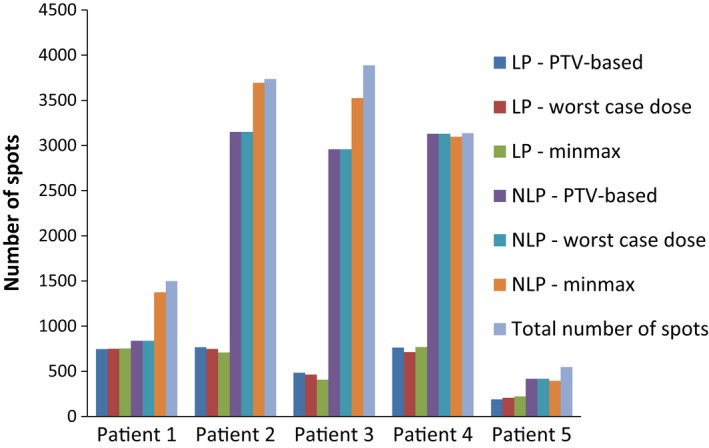
Optimal number of spots resulting from each of the six optimization methods for each patient. Each color represents one method, and the bar on the far right for each patient illustrates the total number of spots in the initial spot arrangement before spot‐intensity optimization.

Next, we determined the resulting number of spots (i.e., spots with positive intensity) for each optimization method. Figure 7 shows the number of spots for the optimal solution for the six methods. For each patient, the bar on the far right in Fig. 7 indicates the total number of spots in the initial spot arrangement. This number was reduced to a smaller number after optimization. In other words, some of the spots were turned off during optimization. We observed many more spots with NLP‐based methods than with LP‐based methods (Table 3).

**Table 3:** Percentages of the total number of spots selected using LP‐ and NLP‐based models averaged for the PTV‐based, worst case dose, and minmax approaches

Model	Percentage of spots selected				
	Patient 1	Patient 2	Patient 3	Patient 4	Patient 5
LP‐based	50	20	12	24	38
NLP‐based	68	89	81	99	75

## 4 Discussion

The inclusion of plan robustness (resilience in the face of uncertainties) in optimization of dose distributions is widely recognized as essential for IMPT. Researchers have developed different planning strategies to account for uncertainties in IMPT planning. In this study, we assessed and compared two mathematical models (LP‐ and NLP‐based) and two robust optimization approaches (worst case dose and minmax) as well as the conventional PTV‐based optimization approach. Our results demonstrated that the robust optimization methods created more robust IMPT plans in terms of target coverage and OAR sparing than did the PTV‐based method. However, the robust optimization methods behaved differently from site to site, and no method emerged as superior to the others under any circumstance.

We performed IMPT planning studies to identify circumstances under which the LP‐ and NLP‐based robust optimization methods behaved differently. It is important to note that the LP‐based models used in this study have hard constraints on dose limits, whereas the NLP‐based models do not. The constrained LP models require model‐specific parameters such as lower and upper dose reference bounds for various organs. These parameters are critical to controlling the outcome of optimization.^17^ However, constrained models have a limitation in the selection of proper values for parameters because if a model is overly constrained (or tightened), it will fail to find a solution that satisfies all user‐defined constraints on organs of interest. Thus, if there is no feasible solution existing for strict model parameter, relaxed parameters must be specified. However, using overly relaxed parameters with a constrained LP‐based model may prevent the model from effectively generating quality treatment plans. We observed that the two LP‐based methods outperformed the two NLP‐based methods in robustness of CTV coverage for patients 1 and 2 (the two prostate cancer cases). We did not observe this difference between the LP‐ and NLP‐based methods for the other three patients. A possible reason for this behavior is that NLP imposes a quadratic penalty on deviations from the desired doses. As a result, NLP‐based models focus more on penalizing large deviations, that is, constraints hard to satisfy or infeasible for LP‐based models, than minor deviations. However, LP‐based models impose a linear penalty on all deviations and focus more on minimizing average overall deviations. In OAR sparing, the performance of both methods was comparable for the first two patients.

**Figure 8:**
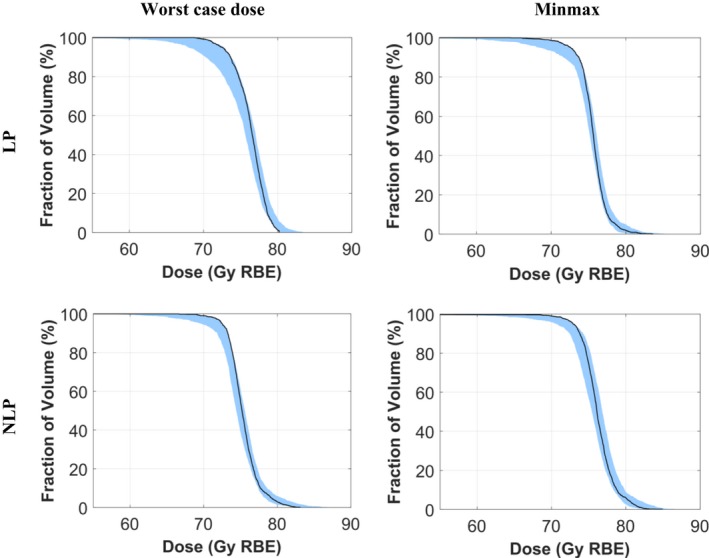
Clinical target volume (CTV) dose‐volume histogram (DVH) bands for dose distributions covering all uncertainties resulting from the use of the four robust optimization methods in patient 5 (with head and neck cancer). The width of the DVH band is inversely proportional to the robustness of the optimization method. The solid lines indicate DVHs for the nominal dose distribution (i.e., without consideration of uncertainties in dose contributions).

Moreover, an optimal dose distribution from a constrained model such as an LP‐based one (see section 2.2) must satisfy all dose‐limit constraints under all possible scenarios. This can be a burden under some scenarios and may result in wider DVH‐family bands than those for an unconstrained model. If a cancer case is not challenging and all scenarios are favorable for finding a feasible solution using constrained LP, the resulting DVH will exhibit better control of plan robustness than that from an unconstrained model, especially for target coverage, as we observed for the two typical prostate cancer cases (see example of CTV coverage and OAR sparing using robust methods for patient 2 in Fig. 3). In comparison, if a cancer case is challenging and some scenarios prevent LP with tight constraints finding a feasible solution, loose upper and lower dose bounds must be used with an LP‐based model and the DVH variations may be large. The complexity of the treatment site and use of very different scenarios are less prominent with an NLP‐based model because NLP does not have hard constraints and the quadratic penalty on the deviation from the target value results in DVH exhibiting less variation than that with LP, which occurred for the skull base and head and neck cancer patients in our study (see examples of CTV coverage with robust methods for patient 5 in Fig. 8), for whom the NLP‐based methods produced more robust plans than did the LP‐based methods.

When comparing worst case dose and minmax methods using either LP or NLP, we found that in the prostate and skull base cancer patients, the worst case dose methods outperformed the minmax methods in terms of robustness of CTV coverage. However, we observed no distinction in the two methods’ robustness in OAR sparing. For the worst case dose methods (voxel‐by‐voxel; see section 2.2.2), the worst case dose of each voxel was calculated independently among all uncertainty scenarios considered. Thus, if one voxel's dose were the worst in one uncertainty scenario, another voxel's dose might be the worst in a different scenario. In other words, all scenarios were used in calculating the objective function value in optimization. However, the minmax methods select one scenario that is the worst case among all scenarios in terms of its objective value; the remaining “easy” scenarios are discarded in the optimization model.^11^ Our results were consistent with those reported by Fredriksson and Bokrantz^11^ for the two typical prostate cancer cases with the same beam arrangement (two lateral opposed beams) and the skull base cancer case, in which the worst case dose methods provided more robust target coverage than did the minmax methods.

We observed a clear difference between the LP‐ and NLP‐based models in terms of the number of spots included in the optimized plans (Table 3). One of the most important features of linear optimization is that the optimal solutions are sparser than those with nonlinear optimization.^18^ As described by Cao et al., LP‐based models can create better dose distributions than NLP‐based models can with only a fraction of the number of prearranged spots required for delivery.^19^ Thus, LP may save IMPT delivery time by reducing the number of scanning spots and, particularly, the number of energy layers.^19^, ^20^, ^21^ It should be noted that we did not see pronounced reduction of energy layers due to the reduction of scanning spots in those test cases in this study. The reduction actual delivery time is therefore only marginal. This indicates that one may have to incorporate specific objective for minimizing the number energy layers in the optimization model in order to reduce delivery time even for LP methods. In addition, LP‐based methods resulted in higher spot intensities (surrogates of monitor units) than did NLP‐based methods as reported by Cao et al.^21^ Therefore, errors in dose calculation resulting from truncation of monitor units to meet the minimum monitor unit constraint can be greatly avoided using LP‐based models.^21^

We also observed that LP‐PTV‐based optimization was outperformed by NLP‐PTV‐based optimization in terms of robustness of CTV coverage in all five cases. The robustness of sparing critical structures for NLP‐PTV‐based optimization was either superior to or comparable with that for LP‐PTV‐based optimization. This may have resulted from the fact that the plans generated using the LP‐based methods were sparser than the plans created using the NLP‐based methods. A sparse solution, in which few spots are selected or have positive intensities, is naturally sensitive to uncertainty if plan robustness is not considered in optimization. However, we did not observe a correlation between the reduction in the number of spots caused by LP‐based robust optimization and loss of robustness. LP‐based robust optimization created considerably more robust plans than did PTV‐based optimization (either LP or NLP) in all cases. It also outperformed NLP‐based robust optimization in plan robustness for the two prostate cancer cases. Overall, plan robustness is primarily determined by whether uncertainty is taken into account in optimization, not by the choice of LP or NLP. Furthermore, whether LP‐ or NLP‐based robust optimization outperforms each other in terms of resulting plan robustness can vary case by case.

A limitation of the robust optimization approaches evaluated in this study was the limited set of predefined uncertainty scenarios. These scenarios accounted only for setup uncertainties along the x, y, and z axes and range uncertainties. We did not consider movements in other directions, nonrigid patient movements, changes in patient anatomy, or other sources of uncertainties. However, incorporating more error scenarios requires more time to solve problems, which may not be practical. Some studies have used a larger number of scenarios.^22^, ^23^ However, evaluation of robustness on the basis of a limited number of setup uncertainties is a common practice and considered to be predictive of both robust optimization and robustness evaluation in IMPT planning.^22^

## 5 Conclusion

The findings of this study reiterate the importance of implementing robust optimization for IMPT. Our results also demonstrate that the robust optimization method ideally should be chosen on a site‐by‐site basis. None of the robust optimization methods (consisting of worst case dose and minmax methods with both LP‐ and NLP‐based models) consistently outperformed the others in terms of either nominal plan quality or plan robustness against uncertainties. The LP‐based methods provided more robust target coverage than did the NLP‐based methods where all uncertainty scenarios were able to meet tight dose constraints, as shown for prostate cancer cases. However, the robustness of the plans created using the LP‐based methods was inferior in more challenging cases, such as head and neck cancer cases, in which some scenarios prevented LP with relatively tighter constraints from finding a feasible solution. In addition, the LP‐based methods resulted in significantly fewer scanning spots than did the NLP‐based methods for the same quality and robustness. With conventional PTV‐based optimization, NLP‐based method outperformed LP‐based method regarding robustness of CTV coverage.

## Conflict of Interest

The authors declare no conflict of interest.

## References

[1] Lomax AJ . Intensity modulation methods for proton radiotherapy. Phys Med Biol. 1999;44:185–205. 10071883 10.1088/0031-9155/44/1/014

[2] Lomax AJ . Intensity modulated proton therapy and its sensitivity to treatment uncertainties 1: the potential effects of calculational uncertainties. Phys Med Biol. 2008;53:1027–1042. 18263956 10.1088/0031-9155/53/4/014

[3] Lomax AJ . Intensity modulated proton therapy and its sensitivity to treatment uncertainties 2: the potential effects of inter‐fraction and interfiled motions. Phys Med Biol. 2008;53:1043–1056. 18263957 10.1088/0031-9155/53/4/015

[4] Pflugfelder D , Wilkens JJ , Oelfke U . Worst case optimization: a method to account for uncertainties in the optimization of intensity modulated proton therapy. Phys Med Biol. 2008;53:1689–1700. 18367797 10.1088/0031-9155/53/6/013

[5] Unkelbach J , Chan TC , Bortfeld T . Accounting for range uncertainties in the optimization of intensity modulated proton therapy. Phys Med Biol. 2007;52:2755–2773. 17473350 10.1088/0031-9155/52/10/009

[6] Unkelbach J , Bortfeld T , Martin BC , Soukup M . Reducing the sensitivity of IMPT treatment plans to setup errors and range uncertainties via probabilistic treatment planning. Med Phys. 2009;36:149–163. 19235384 10.1118/1.3021139 PMC2673668

[7] Liu W , Zhang X , Li Y , Mohan R . Robust optimization in intensity‐modulated proton therapy. Med Phys. 2012;39:1079–1091. 22320818 10.1118/1.3679340 PMC3281975

[8] Chen W , Unkelbach J , Trofimov A , et al. Including robustness in multi‐criteria optimization for intensity‐modulated proton therapy. Phys Med Biol. 2012;57:591–608. 22222720 10.1088/0031-9155/57/3/591 PMC3360481

[9] Fredriksson A , Forsgren A , Hardemark B . Minimax optimization for handling range and setup uncertainties in proton therapy. Med Phys. 2011;38:1672–1684. 21520880 10.1118/1.3556559

[10] Lomax AJ , Pedroni E , Rutz H , Goitein G . The clinical potential of intensity modulated proton therapy. Z Med Phys. 2004;14:147–152. 15462415 10.1078/0939-3889-00217

[11] Fredriksson A , Bokrantz R . A critical evaluation of worst case optimization methods for robust intensity‐modulated proton therapy planning. Med Phys. 2014;41:081701. 25086511 10.1118/1.4883837

[12] Cao W , Lim G , Lee A , et al. Uncertainty incorporated beam angle optimization for IMPT treatment planning. Med Phys. 2012;39:5248–5256. 22894449 10.1118/1.4737870 PMC3422361

[13] Chan TC . Optimization under uncertainty in radiation therapy, Ph.D. Thesis, Operations Research Center, Sloan School of Management, MIT, June 2007.

[14] Fredriksson A . A characterization of robust radiation therapy treatment planning methods‐from 14. expected value to worst case optimization. Med Phys. 2012;39:5169–5181. 22894442 10.1118/1.4737113

[15] Trofimov A , Kang J , Unkelbach J , et al. Evaluation of dosimetric gain and uncertainties in proton therapy delivery with scanned pencil beam in treatment of base‐of‐skull and spinal tumors. Int J Radiat Oncol Biol Phys. 2010;78:S133–S134.

[16] Trofimov A , Unkelbach J , DeLaney TF , Bortfeld T . Visualization of a variety of possible dosimetric outcomes in radiation therapy using dose‐volume histogram bands. Pract Radiat Oncol. 2012;2:164–171. 22773939 10.1016/j.prro.2011.08.001 PMC3388515

[17] Zaghian M , Lim G , Liu W , Mohan R . An automatic approach for satisfying dose‐volume constraints in linear fluence map optimization for IMPT. J Cancer Ther. 2014;5:198–207. 25506501 10.4236/jct.2014.52025 PMC4261934

[18] Candès E , Wakin M , Boyd S . Enhancing sparsity by reweighted l1 minimization. J Fourier Anal Appl. 2008;14:877–905.

[19] Cao W , Lim G , Li X , Li Y , Zhu R , Zhang X . Incorporating deliverable monitor unit constraints into spot intensity optimization in IMPT treatment planning. Phys Med Biol. 2013;58:5113–5125. 23835656 10.1088/0031-9155/58/15/5113 PMC3947922

[20] Kang JH , Wilkens JJ , Oelfke U . Non‐uniform depth scanning for proton therapy systems employing active energy variation. Phys Med Biol. 2008;53:N149. 18401060 10.1088/0031-9155/53/9/N01

[21] Cao W , Lim G , Liao L , et al. Proton energy optimization and reduction for intensity‐modulated proton therapy. Phys Med Biol. 2014;59:6341–6354. 25295881 10.1088/0031-9155/59/21/6341 PMC4371785

[22] Casiraghi M , Albertini F , Lomax AJ . Advantages and limitations of the ‘worst case scenario’ approach in IMPT treatment planning. Phys Med Biol. 2013;58:1323. 23391569 10.1088/0031-9155/58/5/1323

[23] Liu W , Frank SJ , Li X , Li Y , Zhu RX , Mohan R . PTV‐based IMPT optimization incorporating planning risk volumes vs robust optimization. Med Phys. 2013;40:021709. 23387732 10.1118/1.4774363 PMC3562272

